# AI-based hematological malignancy prediction from peripheral blood smears in a large diagnostic laboratory cohort

**DOI:** 10.1038/s41375-026-02934-1

**Published:** 2026-03-23

**Authors:** Muhammed Furkan Dasdelen, Ivan Kukuljan, Peter Lienemann, Fatih Ozlugedik, Ario Sadafi, Matthias Hehr, Karsten Spiekermann, Christian Pohlkamp, Carsten Marr

**Affiliations:** 1 Institute of AI for Health, Helmholtz Zentrum München—German Research Center for Environmental Health, Neuherberg, Germany; 2 International School of Medicine, Istanbul Medipol University, Istanbul, Turkey; 3 Department of Medicine III, Ludwig-Maximilian-University Hospital, Munich, Germany; 4 Munich Center for Machine Learning (MCML), Munich, Germany; 5 Dr. von Haunersches Kinderspital, Ludwig-Maximilians-University Munich, Munich, Germany; 6 DKTK, German Cancer Consortium, Munich, Germany; 7 Munich Leukemia Laboratory, Munich, Germany; 8 Department of Physics, University of Munich, Munich, Germany

**Keywords:** Haematological cancer, Medical research

## Background

Hematological malignancies represent a wide range of disease entities, most of which arise from dysfunctional proliferation and differentiation of hematopoietic stem and progenitor cells in the bone marrow [1]. Diagnosis requires integration of cytomorphology, molecular genetics, and immunophenotyping from blood or bone marrow. Unlike bone marrow aspiration, assessing cytomorphology in a blood smear is fast, minimally invasive, and provides information on differential cell counts and morphological abnormalities that guide follow-up diagnostic pathways. However, conventional peripheral blood smear analysis involves labor-intensive manual examination of hundreds of cells, which is subject to inter-observer variability. Previous work explored machine-learning for single-cell classification [2, 3], and disease detection [4–9] on curated cohorts. Systematic evaluation across multiple malignancies at their natural clinical distribution remains unexplored.

## Methods

**Fig. 1:**
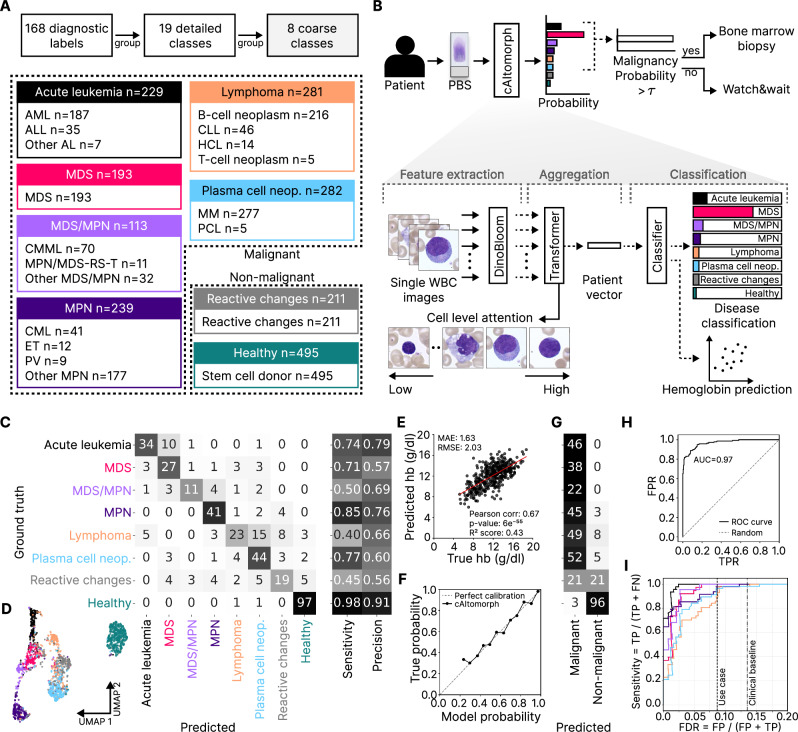
Our diagnostic laboratory dataset contains over 3.2 million single-cell images from blood smears of 6115 patients and 495 healthy stem cell donors with 19 detailed and 8 coarse hematological disease classes. **A** 168 diagnostic labels were hierarchically grouped into 19 detailed classes, which were then further consolidated into broader 8 coarse classes. **B** For routine diagnosis, both peripheral blood smears (PBS) and bone marrow samples were obtained from patients. Cytomorphology, DNA sequencing, immunophenotyping and cytogenetics from bone marrow were used to determine the ground truth label. Model architecture: In the feature extracting stage, the DinoBloom foundation model provides a latent space encoding of each single-cell image. During aggregation, a transformer combines these encodings into a single latent space vector. For classification, we employ a multi-layer perceptron to predict probabilities for the 8 coarse classes and hemoglobin values. We compute the malignancy probability by summing the predicted probabilities of the malignant classes (acute leukemia, MDS, MDS/MPN, MPN, lymphoma, plasma cell neoplasm). If it exceeds the malignancy threshold (0.5 in this study), cAItomorph recommends a bone marrow biopsy. Cell-level attention from the transformer aggregator allow identifying diagnostically relevant cells. **C** Our model distinguishes acute leukemias, MPN and healthy patients with a sensitivity and precision of over 0.74. cAItomorph shows surprisingly high performance on MDS and plasma cell neoplasms. **D** UMAP latent space embeddings show well-distinguishable patient clusters corresponding to different disease classes. Colors correspond to the classes shown in the confusion matrix. **E** Predicted and measured hemoglobin correlate with a Pearson coefficient of 0.67. **F** Model-predicted probabilities align well with true disease distributions, indicating good calibration. **G** Confusion matrix for malignancy vs. non-malignancy predictions at a malignancy threshold of 0.5. **H** cAItomorph achieves 0.97 area under the curve for malignant vs. non-malignant separation. **I** By tuning cAItomorph’s malignancy threshold, we can reduce the rate of unnecessary bone marrow aspirations (FDR) from 13.5% (Clinical baseline) to 8.7% while maintaining 100% sensitivity for acute leukemia.

We retrospectively collected digitized white blood cell images from peripheral blood smears from 6610 individuals (6115 patients and 495 healthy stem cell donors) processed at the Munich Leukemia Laboratory (MLL) between 2021 and 2022, encompassing the full spectrum of hematological malignancies. We grouped 168 MLL diagnostic labels into 19 detailed and 8 coarse classes (Fig. 1A). To develop a model for initial diagnosis, we excluded post-chemotherapy patients and ended up with 1634 for model training and 409 for internal testing (Supplementary Fig. 1A). We also tested the model on 1408 patients with unclear diagnoses or borderline classifications.

Using the DinoBloom foundation model [10], we trained a transformer-based AI model, cAItomorph, to predict the coarse classes of hematological malignancies from peripheral blood smear single-cell images (Fig. 1B). cAItomorph consists of three steps: (i) the DinoBloom encoder extracts 768 features from individual white blood cell images. (ii) A transformer [11] aggregator combines the information from 500 cells into a single 512-dimensional representation. (iii) A classifier outputs probabilities for each of the eight classes. Using 5-fold cross-validation, we trained five models and combined their predictions via ensembling testing on a held-out test set (see Supplementary Methods and Supplementary Fig. 1B).

## Results

Our model achieves a high sensitivity and precision for acute leukemia (sensitivity/precision=0.74/0.79) and MPN (0.85/0.76), and a moderate performance for MDS (0.71/0.57) (Fig. 1C). Surprisingly, cAItomorph achieves high sensitivity on plasma cell neoplasms (0.77) despite the fact that these are typically not detectable in blood smears. For lymphoma, the sensitivity is expectedly low (0.40/0.66), as well as for MDS/MPN (0.50/0.69) and reactive changes (0.45/0.56). These disease classes are notoriously difficult to diagnose from peripheral blood. The overall accuracy of the model is 0.72. Two-dimensional UMAP projections of patient embeddings reveal clusters corresponding to coarse disease classes, with a clear split between myeloid and lymphoid branches, and a separation of healthy individuals (Fig. 1D). As an auxiliary task, the model predicts hemoglobin values, which correlate significantly with measured values (Pearson correlation coefficient 0.67, *p* = 6 × 10^−55^; mean absolute error (MAE) 1.63; Fig. 1E). cAItomorph’s output probabilities match true disease probabilities (expected calibration error of 2.4%, Fig. 1F), ensuring reliable confidence in predictions. The model achieves top-2 accuracy of 0.87 (Supplementary Fig. 2).

By summing the probabilities of all malignant class predictions, our model can predict malignancy and thus guide further testing (Fig. 1B). For binary malignant vs. non-malignant prediction (Fig. 1G), our model achieves a sensitivity of 0.94 and specificity of 0.83, with an AUC of 0.97 (Fig. 1H). In our cohort, 13.5% of patients who underwent bone marrow aspiration were subsequently diagnosed with reactive changes, representing potentially avoidable invasive procedures. By adjusting the model’s malignancy threshold, we can balance between detecting every leukemia case and avoiding unnecessary bone marrow aspirations. With a 0.5 malignancy threshold, we can reduce the rate of unnecessary bone marrow aspirations from 13.5% to 8.7% (35% relative reduction) while correctly identifying all 46 acute leukemia cases in the test set (Fig. 1I).

**Fig. 2:**
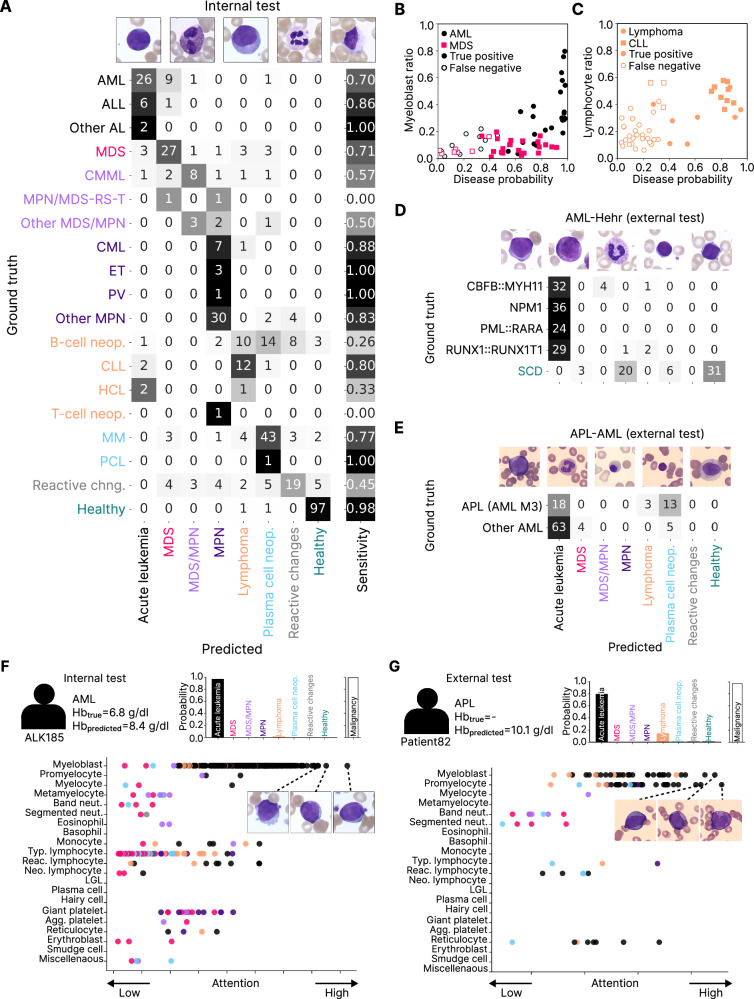
cAItomorph predicts hematological malignancies from peripheral blood images in internal and external datasets while highlighting clinically relevant cells. **A** cAItomorph achieves high sensitivities in subtypes with visible aberrations or high cellular counts, such as AML, ALL, CLL, ET, and CML. cAItomorph also achieves high sensitivity on multiple myeloma cases, although it is not typically diagnosed from morphology. As expected, performance was lower in disease classes not diagnosed from peripheral blood, such as lymphoma (excluding CLL). The model almost perfectly identified healthy donors, but, as expected, it struggles with patients exhibiting reactive changes. **B** cAItomorph acute leukemia prediction probability correlates with myeloblast ratio in the blood. Acute leukemia patients with a low myeloblast ratio are misclassified as MDS. **C** cAItomorph lymphoma prediction probability correlates with lymphocyte ratio in the blood. The cases with a high lymphocyte ratio belong to the CLL subtype. **D** Model performance on the AML-Hehr dataset. Ground-truth labels include four genetic subtypes of AML and stem cell donors (SCD). cAItomorph identifies all acute leukemia cases with high sensitivity and precision. **E** Confusion matrix for predictions on the APL-AML dataset. **F** Two exemplary patients are presented: one from the internal test set and one from the external APL-AML dataset. For each patient, we provide metadata, predicted hemoglobin, eight-class hematological disease probabilities, malignancy probability, and attention distribution over cells. Individual cells are passed through cAItomorph to better understand their relation to diseases. Cell dots are colored based on model predictions. An AML patient from the internal test set was confidently predicted as acute leukemia by cAItomorph. Myeloblasts receive the highest attention. **G** An APL patient from the APL-AML dataset. Promyelocytes and myeloblasts receive the highest attention, as expected.

We next analysed cAItomorph’s prediction capabilities with respect to the 19 detailed classes. Our model achieved a 0.70 sensitivity on acute myeloid leukemia (AML) cases, which present with myeloblasts in peripheral blood and are thus easier to identify compared to other classes, assigning 9 out of 37 AML cases to MDS (Fig. 2A). To analyse the misclassified cases, we plot the measured myeloblast ratio vs. the disease probability (Fig. 2B). cAItomorph’s AML disease probability correlates with the myeloblast ratio in the blood (Spearman correlation coefficient 0.73, *p* = 7 × 10^–7^). All misclassified cases have a myeloblast ratio < 20% and are assigned as MDS.

Myeloproliferative neoplasms (MPN) are clonal disorders characterized by proliferation of one or more myeloid lineages, resulting in mature cell overproduction. Our model correctly classifies 11 out of 12 cases with essential thrombocytosis (ET), polycythemia vera (PV), and chronic myeloid leukemia (CML), and achieved 0.83 sensitivity in other MPN cases. In contrast, MDS is characterized by varying degrees of single or multiple cytopenias [1] and dysplasia. cAItomorph effectively distinguishes these differences from peripheral blood cell images, misclassifying only 1 out of 38 MDS cases as MPN (Fig. 2A). However, there is an expected confusion with MDS/MPN cases, rare clonal bone marrow disorders with overlapping features of both MDS and MPN: 50.0% are predicted correctly; 13.6% are predicted as MDS, and 18.2% are predicted as MPN (Fig. 2A).

For lymphoid malignancies, 12 out of 15 CLL cases are correctly classified (0.80 sensitivity), but 26% of lymphoma cases are misclassified as plasma cell neoplasm, predominantly B-cell neoplasms (Fig. 2A). By plotting lymphocyte ratio in blood vs. lymphoma model probability, we find that the model detects lymphoma cases more accurately when lymphocyte counts are elevated (Fig. 2C). Notably, most of these cases belong to the CLL subtype.

We further evaluate our model on an extended internal test set including patients with unclear/rare diagnoses, double diagnoses, and MGUS cases previously excluded (Supplementary Fig. 4A). Our model is able to classify borderline cases into one of the suspected classes. For instance, out of 11 ‘MDS-AML borderline’ cases, 5 are assigned to acute leukemia and 4 are assigned to MDS; out of 4 ‘MPN in blast crisis’ cases, 3 are assigned to acute leukemia, and 1 to MPN. Notably, cAItomorph classifies 77 out of 199 MGUS cases as plasma cell neoplasms (39%), which is a precursor condition of multiple myeloma. Predicted hemoglobin values correlate well with measured values (Pearson correlation coefficient 0.66, Supplementary Fig. 4B).

We evaluate cAItomorph’s generalizability on two external datasets: AML-Hehr [4] includes four genetic AML subtypes and healthy stem cell donors, while APL-AML [7] comprises only AML patients, grouped into acute promyelocytic leukemia (APL) and other AML subtypes. In AML-Hehr, our model misclassifies only 8 out of 129 AML patients, achieving a sensitivity of 0.94 for acute leukemias and an AUROC of 0.99 in distinguishing malignant from non-malignant samples (Fig. 2D, Supplementary Fig. 3A). For APL-AML, having a distinct staining background, cAItomorph achieves a 0.76 sensitivity for detecting acute leukemias and a 1.0 sensitivity for malignancy detection (Fig. 2E). Notably, external cases aligned closely with internal test samples in a low-dimensional UMAP embedding, forming coherent clusters consistent with disease types (Supplementary Fig. 3B).

To illustrate clinical utility, we visualize cell-level attentions for representative cases in an interactive dashboard at https://github.com/marrlab/cAItomorph. The model successfully assigns high attention to myeloblasts when classifying an acute leukemia case, while assigning minimal attention to typical lymphocytes (Fig. 2F). In an APL patient from the external APL-AML dataset, cAItomorph correctly highlights promyelocytes and myeloblasts (Fig. 2G). In a patient with myeloproliferative neoplasm (MPN), the model highlights giant platelets that can hint at the diagnosis of MPN (Supplementary Fig. 5A). In a patient with *CBFB::MYH11* fusion from the external AML-Hehr dataset, the model identifies myeloblasts and monocytic cells, consistent with the monocytic differentiation typical of this AML subtype (Supplementary Fig. 5B).

## Conclusion

We developed cAItomorph, a transformer-based model for multi-disease classification of hematological malignancies from peripheral blood smears. In a diagnostic laboratory cohort, the model achieved high accuracy for acute leukemias, myeloproliferative neoplasms, and healthy controls, with robust performance on external validation cohorts. Based on test-set performance, the model may help reduce unnecessary bone marrow aspirations (by up to 35% in our test set) while preserving sensitivity for acute leukemia, although prospective validation is needed. Notably, image-based diagnostics outperformed models based on differential cell counts and demographic variables (age and sex; Supplementary Table 3).

Important limitations are the retrospective, single-center study design, class imbalance affecting the detection of rare diseases, and a healthy control group limited to stem cell donors. Prospective multi-center validation is required to assess real-world clinical utility. Nevertheless, cAItomorph demonstrates the feasibility of AI-based peripheral blood screening as a complementary tool for initial diagnostic triage in hematological malignancies, potentially reducing the burden of invasive procedures while maintaining diagnostic sensitivity.

## Supplementary information

Supplementary material

## Data Availability

We release the test set of our comprehensive peripheral blood cytomorphology image dataset. This data encompasses 409 individuals, categorized as follows: 46 acute leukemia, 38 MDS, 22 MDS/MPN, 48 MPN, 57 lymphoma, 57 plasma cell neoplasm, 42 reactive changes and 99 healthy. The dataset covers the entire spectrum of hematological malignancies, reflecting their relative frequencies within the population. Altogether, it consists of 201,560 blood cell images. The test dataset is available at 10.82296/hmgu-nefeli.9bv4e-3ag16. External test sets are available under the corresponding links: AML-Hehr: https://www.cancerimagingarchive.net/collection/aml-cytomorphology_mll_helmholtz/. APL-AML: https://www.kaggle.com/datasets/eugeneshenderov/acute-promyelocytic-leukemia-apl.

## Code availability

Our code and model weights are available at https://github.com/marrlab/cAItomorph.
