# Supplementary material for: AI-based hematological malignancy prediction from peripheral blood smears in a large diagnostic laboratory cohort
Source: Leukemia. 2026 Mar 23;40(6):1318–22. doi: 10.1038/s41375-026-02934-1 (PMC13233331; doi:10.1038/s41375-026-02934-1)
Supplement: Supplementary file 1 — Supplementary material [file 41375_2026_2934_MOESM1_ESM.pdf]

AI-Based Hematological Malignancy Prediction from Peripheral Blood Smears in a Large Diagnostic Laboratory Cohort

Muhammed Furkan Dasdelen[1,2,*], Ivan Kukuljan[1,*], Peter Lienemann[1,3,], Fatih Ozlugedik[1], Ario Sadafi[1], Matthias Hehr[1,4], Karsten Spiekermann[3], Christian Pohlkamp[5], and Carsten Marr[1,3,6,7,8]

[1]Institute of AI for Health, Helmholtz Zentrum München—German Research Center for Environmental Health, Neuherberg, Germany

[2]International School of Medicine, Istanbul Medipol University, Istanbul, Turkiye

[3]Department of Medicine III, Ludwig-Maximilian-University Hospital, Munich, Germany

[4]Dr. von Haunersches Kinderspital, Ludwig-Maximilians-University Munich, Munich, Germany

[5]Munich Leukemia Laboratory, Munich, Germany

[6]DKTK, German Cancer Consortium, Munich, Germany

[7]Munich Center for Machine Learning (MCML), Munich, Germany

[8]Department of Physics, University of Munich, Munich, Germany

*These authors contributed equally to this work

Correspondence: carsten.marr@helmholtz-munich.de

Supplementary methods

Data

Our diagnostic laboratory cohort comprises 6115 patients diagnosed for the first time at Munich Leukemia Laboratory (MLL) during the years 2021-2022, along with 495 healthy donors (**Supplementary Fig. 1A**). Peripheral blood and bone marrow samples were obtained during routine diagnostic workup. Diagnoses were made based on bone marrow cytomorphology, immunophenotyping, and cyto- and molecular genetics, as defined by the WHO guidelines[1]. These diagnoses served as the ground truth labels. This study involved retrospective analysis of routine diagnostic samples; no patients were prospectively recruited. The requirement for informed consent was waived due to the retrospective nature of the analysis using archival peripheral blood smears. The retrospective analysis of images received approval from the Ethics Committee of the Medical Faculty, Ludwig-Maximilians-Universität (LMU) Munich, Germany (Approval No. 25-0744).

Single white blood cell images were obtained as described previously[2]. Briefly, Wright-Giemsa stained peripheral blood smears were initially scanned using a 10x objective, producing an overview image. The Metasystems Metafer software then detected high quality single leukocyte images after a segmentation threshold. The largest possible number of leukocytes with sufficient quality were positioned in each image and scanned with a 40x objective. Images were stored in TIFF format with 144x144 pixels. Additionally, basic information about the patients (age, sex, and blood counts) is available.

Data cleaning and diagnostic label grouping

We cleaned the dataset by excluding patients with post-chemotherapy/treatment follow-ups (n=3159), patients with insufficient cell counts (n=9), MGUS (n=216), double or in-between diagnosis (n=202), rare conditions (n=52) and unclear diagnosis (n=929) (**Supplementary Fig. 1A**). Parts of this excluded data were reintroduced later in the extended test set (see Results). After cleaning, our dataset comprised 2043 patients with a total of 996,087 single-cell images. The final cleaned data includes 478±55 single white blood cell images per patient.

The dataset contains 168 different diagnosis labels, some common and some rare. We hierarchically grouped the labels into 19 detailed classes, such as "AML" (including subtypes), "B-cell neoplasm", or "CMML". We then grouped diseases into 8 coarse classes, namely "Acute leukemia", "Lymphoma", "MDS", "MDS/MPN", “MPN”, “Plasma cell neoplasm” , “Reactive changes” and “Healthy” (**Fig. 1A**).

We reserved 20% of the data (409 cases) for testing by stratifying according to detailed classes.

AI architecture and model training

The task involves assessing ~500 single leukocyte images per patient, and determining the correct patient level diagnosis. This constitutes a weakly supervised learning problem and has been previously applied in hematology.[2,4–6] Our models consist of three steps: (i) Latent space encoding of single-cell images, (ii) feature vector aggregation, and (iii) classification (**Fig. 1B**). The goal of encoding is to compress single-cell images into a 768 dimensional feature vector. In the second step, we aggregate the feature vectors from all white blood cells into one single 512 dimension vector. This vector is then utilized by a classifier to predict the diagnosis.

For the encoding part, we use the DinoBloom hematology foundation model (ViT-B)[7] with 86M parameters. For the aggregation, we use a modified vision transformer architecture (ViT)[8,9] with 10 transformer layers and 8 attention heads of dimension 64, resulting in an embedding dimension of 512, and a hidden dimension of 2,048. We ablate the number of layers and token pooling strategy and compare to other multiple instance learning aggregators (**Supplementary Table 1, 2**). The classifier is a 2-layer MLP (multi-layer perceptron) with a 128-dimensional hidden layer.

The model is trained with AdamW optimizer, weight decay of 0.01, learning rate of 5e-5 for 150 epochs with early stopping based on validation loss. We use cross entropy loss for disease classification and mean square error for hemoglobin prediction.

Model ensembling

For model development, we conduct 5-fold cross-validation. The softmax function is applied to the output logits to obtain class probabilities. During testing, predictions from the five models were averaged to produce ensemble outputs (**Supplementary Fig. 1B**). To derive a patient’s overall malignancy probability, we sum up the probabilities corresponding to Acute leukemia, Lymphoma, MDS, MDS/MPN, MPN, and Plasma cell neoplasm classes (**Fig. 1B**).

Explainability

Explainability is crucial to ensure that an algorithm bases its decisions on relevant features within the data. We obtain cell level attentions from transformer heads using the Attention Rollout method[10]. This method provides insights into how each image contributes to the diagnostic decision.

External datasets

We test our model on two external datasets. AML-Hehr[2] includes 129 patients diagnosed with four prevalent AML subtypes with defining genetic abnormalities and 60 healthy stem cell donors. The dataset consists of 430±107 single white blood cell images per patient. APL-AML[3] has 106 patients, all diagnosed with acute leukemia and categorized into two: acute promyelocytic leukemia (APL) and other AML subtypes.

Evaluation metrics

For model performance evaluation, we calculate sensitivity, precision, and F1 score. Sensitivity (recall) is the proportion of true positive cases among all positive cases. Precision is the ratio of true positive cases among all predicted positive cases. The F1 score is the harmonic mean of sensitivity and precision. We also measure the false discovery rate, which is calculated as 1 - precision, representing the proportion of false positive predictions among all positive predictions made by the model.

Supplementary Figures

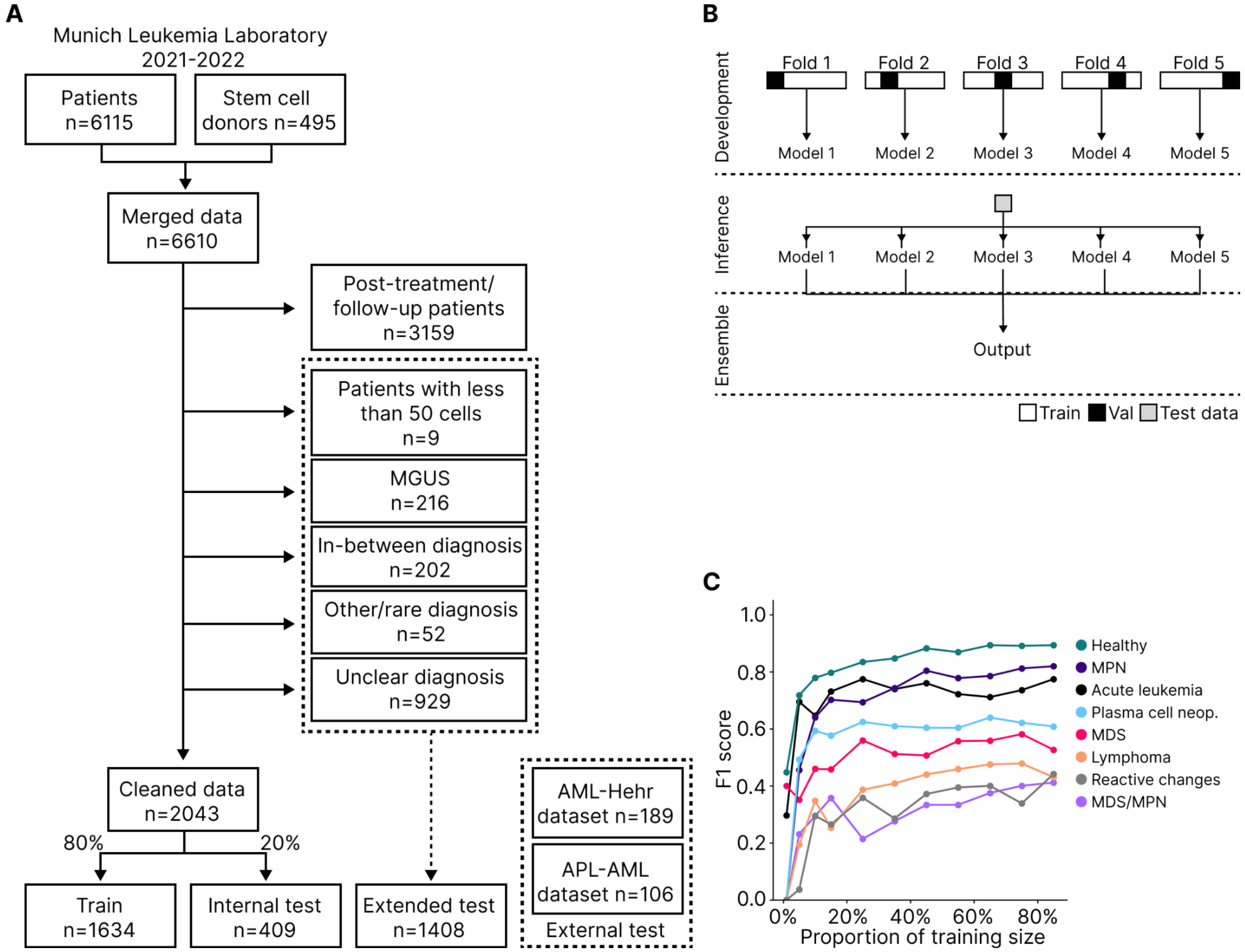

Supplementary Figure 1. Data collection and model training. (A) We retrospectively collected peripheral blood samples from 6115 patients and 495 healthy donors (healthy) processed at the Munich Leukemia Laboratory (2021–2022) (total n=6610). To focus on initial diagnosis, we excluded post-treatment and follow-up samples (n=3159). Samples with <50 evaluable cells, MGUS, double or in-between diagnoses, other/rare diagnosis, and unclear diagnosis were not used for model development and were instead grouped as an extended test set (n=1408). The remaining cleaned dataset comprised n=2043 samples and was split into training (80%, n=1634) and an internal test set (20%, n=409). We further evaluated generalization on external cohorts (AML-Hehr, n=189; APL-AML, n=106). **(B)** Within the training split (n=1634), we performed 5-fold cross-validation, training five models. At inference time, each sample was evaluated by all five models and the predicted class probabilities were averaged to obtain the final disease probabilities (ensemble). **(C)** Model performance plateaus at ~50% of the training data, confirming sufficient dataset size for the model architecture.

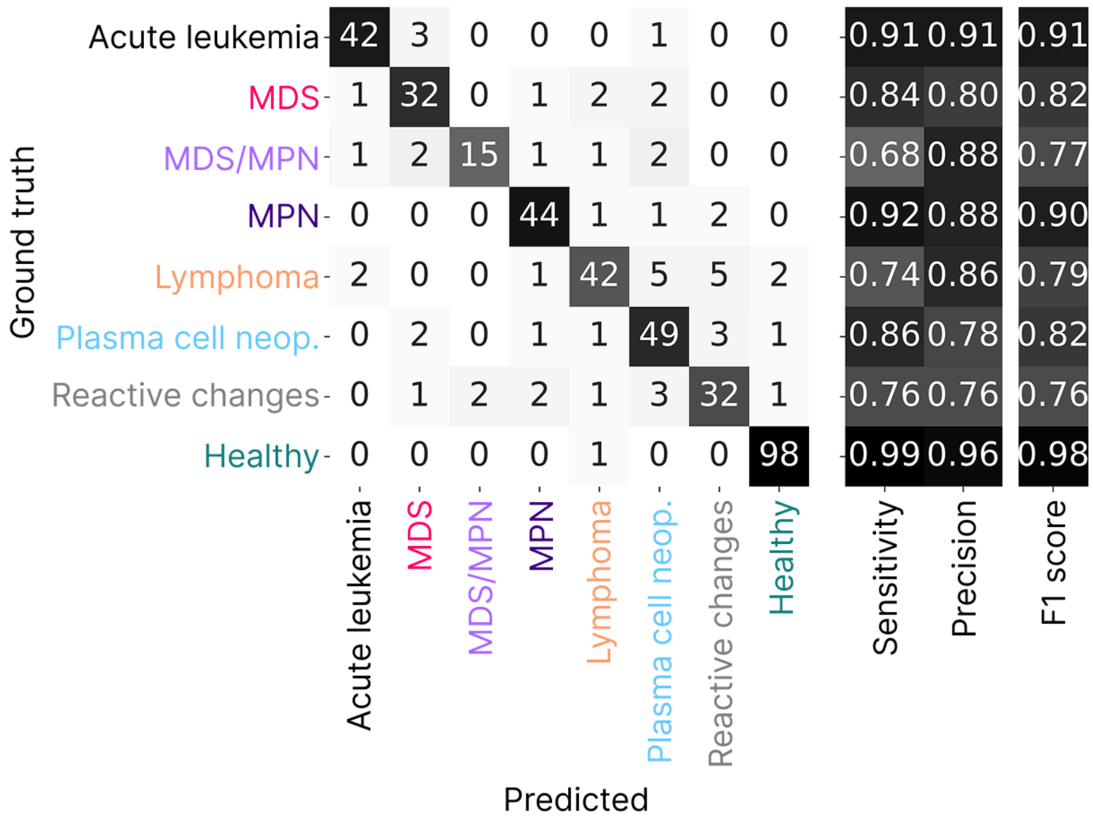

Supplementary Figure 2. Model performance improves at top-2 prediction, especially for lymphoma, MDS/MPN, and reactive changes. (A) We analyzed the model's performance for the top-2 prediction, where a prediction is considered correct if the correct label is among the two classes with the highest probabilities. Overall accuracy of the model improves from 0.72 to 0.87 at top-2 prediction.

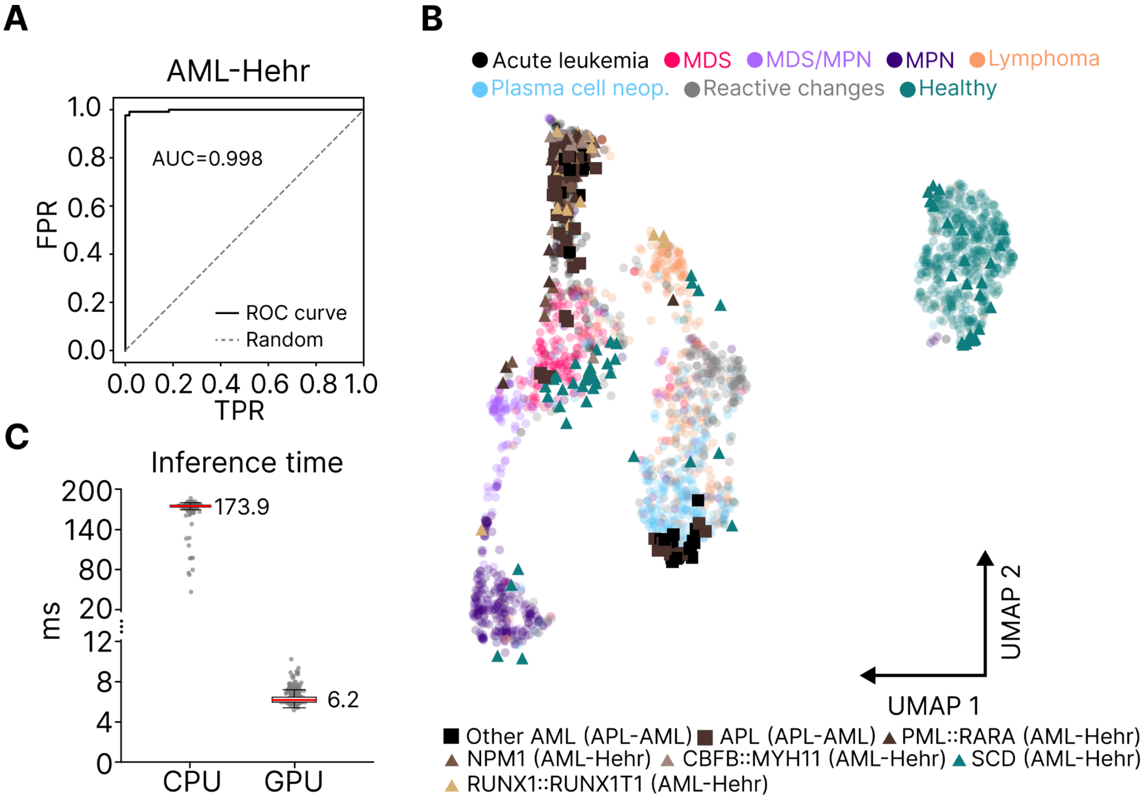

Supplementary Figure 3. cAItomorph generalizes to external cohorts and separates malignant cases. **(A)** In the AML-Hehr external dataset, cAItomorph distinguishes malignant vs. non-malignant cases with AUC = 0.99. **(B)** When projected into the model embedding space and visualized with UMAP, external cohort samples (squares for APL-AML dataset, triangles for AML-Hehr dataset) co-localize with the corresponding internal diagnostic clusters (circles), indicating consistent representation learning. **(C)** Per-sample inference time on a single CPU vs. GPU (NVIDIA H100 80GB). The median inference time is 173.9 ms on the CPU, compared to 6.2 ms on the GPU.

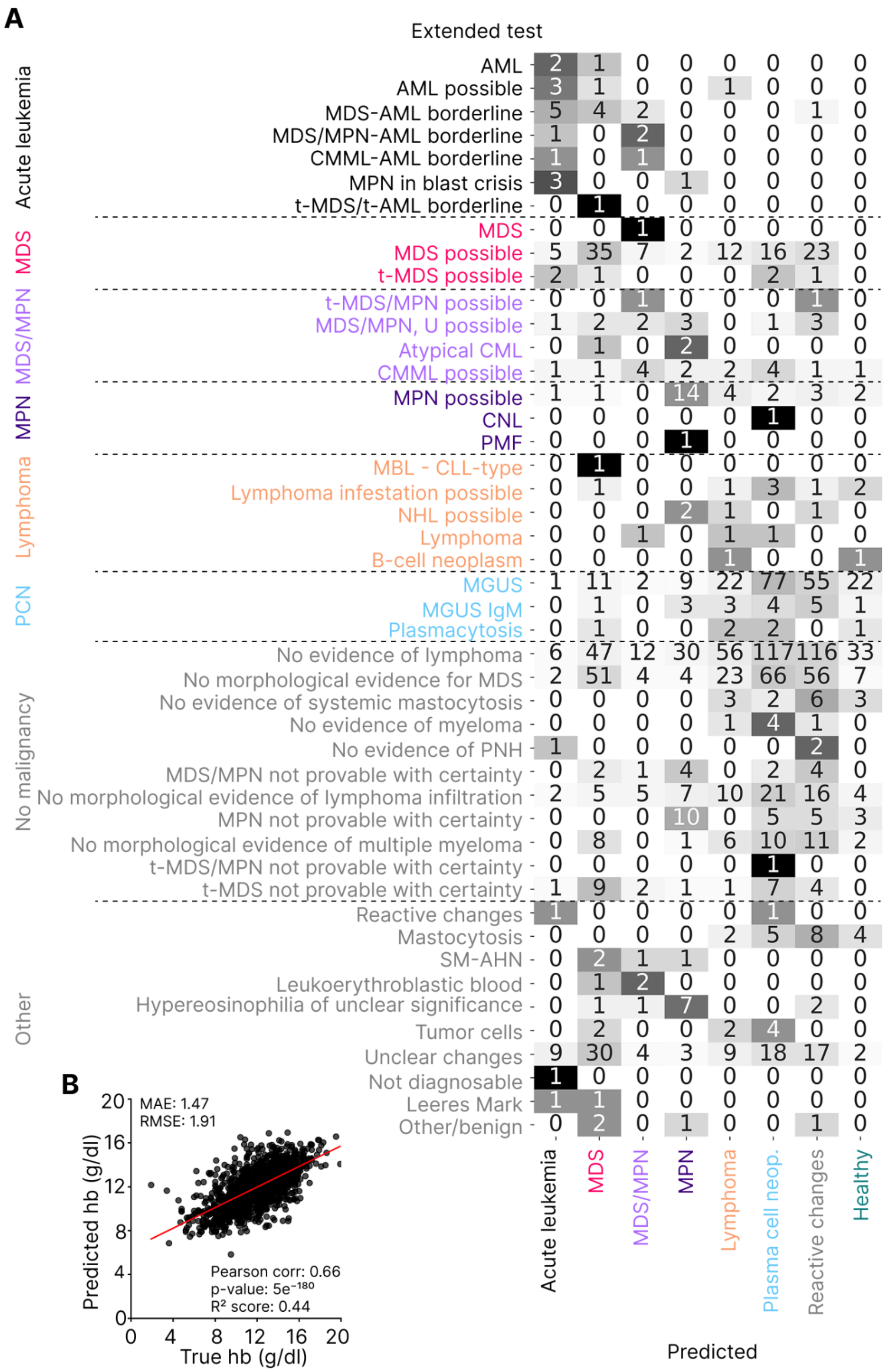

Supplementary Figure 4. Extended internal test set performance. (A) Borderline cases are successfully assigned into one of the suspected classes. For instance, 5/11 assigned as acute leukemia and 4/11 assigned as MDS in MDS-AML borderline cases. MPN in blast crises are assigned either in acute leukemia (3/4) or MPN (1/4). Notably, cAItomorph classifies 38% of MGUS cases as plasma cell neoplasms, which is a precursor condition of multiple myeloma. **(B)** Predicted hemoglobin (hb) and measured true hemoglobin values correlates well with a Pearson coefficient of 0.66.

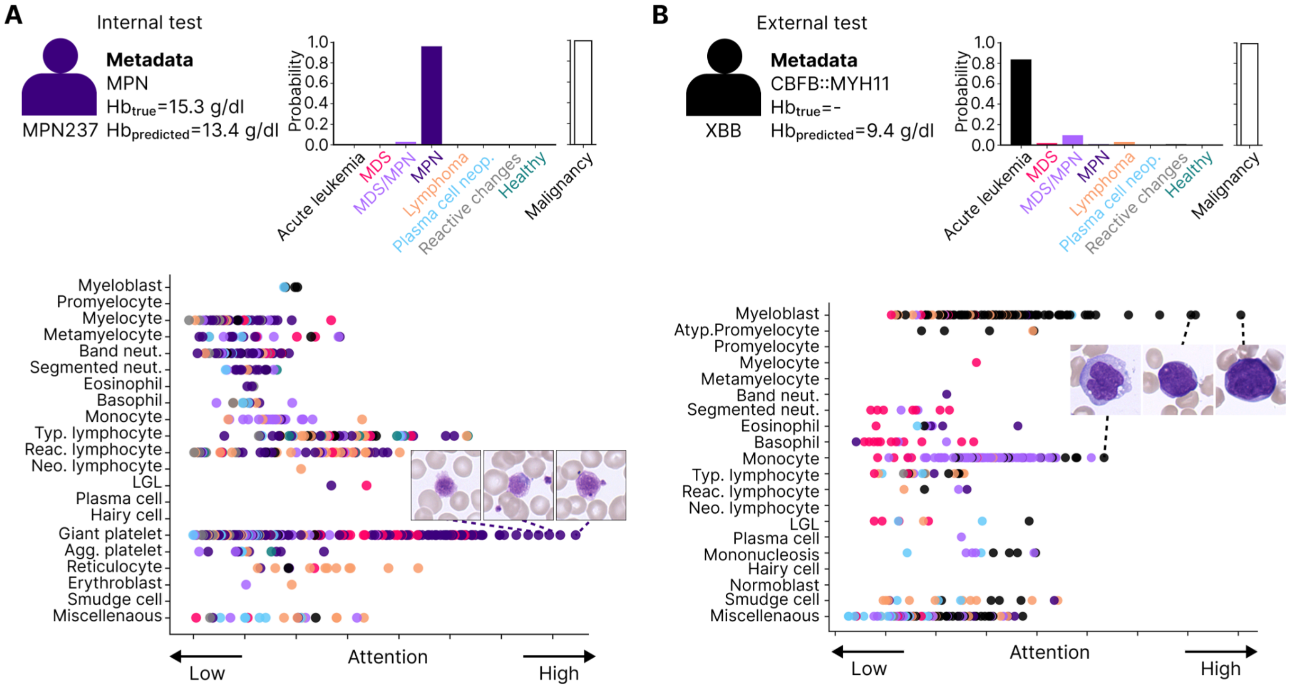

Supplementary Figure 5. (A) An MPN patient from the internal test set, predicted to be an MPN and malignant. cAItomorph assigns high attention to giant thrombocytes, which supports the diagnosis of MPN. Predicted hemoglobin values correlate with the magnitude of the actual measured values, although they do not match exactly. **(B)** An AML case with a CBFB::MYH11 fusion mutation from the AML-Hehr dataset. cAItomorph diagnoses the case with high confidence while assigning high attention to myeloblasts and monocytic cells.

Ablation Studies

Supplementary Table 1. Comparison of different aggregator models. A transformer-based architecture performs best compared to other methods.

	Balanced accuracy	Weighted F1
Transformer[8]	**0.64±0.03**	**0.68±0.03**
WBCMIL[2]	0.60±0.02	0.65±0.01
ABMIL[11]	0.52±0.01	0.57±0.01
Mean	0.60±0.02	0.65±0.02

Supplementary Table 2. Ablation on different pooling strategies in the transformer model and number of transformer layers. * denotes the architecture used in this study.

Pooling	Depth	Balanced accuracy	Weighted F1
CLS pooling			
CLS	2	0.61±0.02	0.66±0.03
CLS	4	0.61±0.01	0.66±0.01
CLS	6	0.62±0.02	0.67±0.03
CLS	8	0.63±0.02	0.68±0.02
*CLS	10	0.64±0.03	0.68±0.03
CLS	12	0.63±0.02	0.67±0.02
Mean pooling			
Mean	2	0.63±0.02	0.68±0.02
Mean	4	0.64±0.02	0.68±0.02
Mean	6	0.63±0.02	0.68±0.01
Mean	8	0.64±0.01	0.68±0.02
Mean	10	0.62±0.02	0.67±0.02
Mean	12	0.63±0.01	0.68±0.01

Supplementary Table 3 | Performance of demographic variables and differential blood counts compared with peripheral blood smear images (cAItomorph). We evaluate how well simple clinical metadata (age, sex, and differential blood counts) predict diagnosis compared with the image-based cAItomorph model. Differential blood counts quantify the relative composition of cell types in the peripheral blood smear, including myeloblasts, promyelocytes, myelocytes, metamyelocytes, band neutrophils, segmented neutrophils, eosinophils, basophils, monocytes, small lymphocytes, large lymphocytes, and neoplastic lymphocytes. For a fair ablation, all non-image models use the same classifier architecture as cAItomorph, i.e., a multilayer perceptron (MLP) with 128 hidden dimensions, trained with identical hyperparameters across settings. Results are reported as mean ± s.d. across splits. Differential blood counts alone provide moderate discriminative signal, and combining them with age improves performance, but the full image-based cAItomorph model achieves the best overall balanced accuracy and weighted F1.

	Balanced accuracy	Weighted F1
Age only	0.27±0.01	0.31±0.01
Sex only	0.14±0.01	0.12±0.02
Blood count only	0.43±0.01	0.46±0.01
Age + sex	0.26±0.02	0.31±0.01
Age + blood count	0.52±0.02	0.58±0.01
Sex + blood count	0.44±0.01	0.47±0.01
Age + sex + blood count	<u>0.53±0.01</u>	<u>0.58±0.01</u>
Peripheral blood images (cAItomorph)	**0.64±0.03**	**0.68±0.03**

Supplemental References

1 Khoury JD, Solary E, Abla O, Akkari Y, Alaggio R, Apperley JF *et al.* The 5th edition of the World Health Organization Classification of Haematolymphoid Tumours: Myeloid and Histiocytic/Dendritic Neoplasms. *Leukemia* 2022; **36**: 1703–1719.

2 Hehr M, Sadafi A, Matek C, Lienemann P, Pohlkamp C, Haferlach T *et al.* Explainable AI identifies diagnostic cells of genetic AML subtypes. *PLOS Digit Health* 2023; **2**: e0000187.

3 Sidhom J-W, Siddarthan IJ, Lai B-S, Luo A, Hambley BC, Bynum J *et al.* Deep learning for diagnosis of acute promyelocytic leukemia via recognition of genomically imprinted morphologic features. *NPJ Precis Oncol* 2021; **5**: 38.

4 Sadafi A, Adonkina O, Khakzar A, Lienemann P, Hehr RM, Rueckert D *et al.* Pixel-Level Explanation of Multiple Instance Learning Models in Biomedical Single Cell Images. In: *Information Processing in Medical Imaging*. Springer Nature Switzerland, 2023, pp 170–182.

5 Kazeminia S, Sadafi A, Makhro A, Bogdanova A, Albarqouni S, Marr C. Anomaly-Aware Multiple Instance Learning for Rare Anemia Disorder Classification. In: *Medical Image Computing and Computer Assisted Intervention – MICCAI 2022*. Springer Nature Switzerland, 2022, pp 341–350.

6 Sadafi A, Makhro A, Bogdanova A, Navab N, Peng T, Albarqouni S *et al.* Attention Based Multiple Instance Learning for Classification of Blood Cell Disorders. In: *Medical Image Computing and Computer Assisted Intervention – MICCAI 2020*. Springer International Publishing, 2020, pp 246–256.

7 Koch V, Wagner SJ, Kazeminia S, Sancar E, Hehr M, Schnabel J *et al.* DinoBloom: A Foundation Model for Generalizable Cell Embeddings in Hematology. arXiv [cs.CV]. 2024.http://arxiv.org/abs/2404.05022.

8 Dosovitskiy A, Beyer L, Kolesnikov A, Weissenborn D, Zhai X, Unterthiner T *et al.* An Image is Worth 16x16 Words: Transformers for Image Recognition at Scale. arXiv [cs.CV]. 2020.http://arxiv.org/abs/2010.11929.

9 Ding T, Wagner SJ, Song AH, Chen RJ, Lu MY, Zhang A *et al.* A multimodal whole-slide foundation model for pathology. *Nat Med* 2025; **31**: 3749–3761.

10 Abnar S, Zuidema W. Quantifying Attention Flow in Transformers. arXiv [cs.LG]. 2020.http://arxiv.org/abs/2005.00928.

11 Ilse M, Tomczak J, Welling M. Attention-based Deep Multiple Instance Learning. In: *International Conference on Machine Learning*. PMLR, 2018, pp 2127–2136.
